# Supplementary material for: Time‑resolved multi-omic analysis of paclitaxel exposure in human iPSC‑derived sensory neurons unveils mechanisms of chemotherapy‑induced peripheral neuropathy
Source: Cell Death Dis. 2026 Feb 10;17(1):211. doi: 10.1038/s41419-026-08445-2 (PMC12921266; doi:10.1038/s41419-026-08445-2)
Supplement: Supplementary file 15 — Dataset 1. [file 41419_2026_8445_MOESM15_ESM.zip › CDD_Open_Data/05_Protein/Archive/01_string_vector_graphic.docx]

**Basic Settings**

Network type:

full STRING network

( the edges indicate both functional and physical protein associations )

physical subnetwork

( the edges indicate that the proteins are part of a physical complex )

meaning of network edges:

evidence

(   line color indicates the type of interaction evidence )

confidence

(   line thickness indicates the strength of data support )

active interaction sources:

Textmining

Experiments

Databases

Co‑expression

Neighborhood

Gene Fusion

Co‑occurrence

minimum required interaction score:

highest confidence (0.900)high confidence (0.700)medium confidence (0.400)low confidence (0.150)custom value

max number of interactors to show:

1st shell:

- none / query proteins only -no more than 5 interactorsno more than 10 interactorsno more than 20 interactorsno more than 50 interactorscustom value

2nd shell:

- none -no more than 5 interactorsno more than 10 interactorsno more than 20 interactorsno more than 50 interactors  custom value

[**UPDATE**](javascript:document.getElementById('standard_parameters').submit();)

**Advanced Settings**

network display mode:

static png

(   network is a simple bitmap image; not interactive )

interactive svg

(   network is a scalable vector graphic [SVG]; interactive )

network display options:

Enable node coloring mode

enable 3D bubble design

disable structure previews inside network bubbles

center protein names on nodes

show your query protein names

hide disconnected nodes in the network

hide protein names

 protein name font size

**Proteins (50 best correlated genes with proteins)**

HMGCR

MMP10

SCD

JUN

SESN2

ATF3

TNFRSF12A

MSMO1

FADS2

SQLE

HMGCS1

FDFT1

NEDD4

CALCB

SOX11

DHCR24

TMEM97

ITGB8

PPP1R17

IDI1

LSS

WEE1

ABCB1

PIRT

GBP2

SC5D

LRP4

MYPN

LDLR

ARHGEF28

LDLR

ULBP1

STMN4

CCDC184

SMAD1

RNF145

WNT11

NES

SLC1A3

HEG1

CASP3

FJX1

DIO3

SPCS3

FAM53C

IQGAP3

IGFBPL1
